# Is Artificial Intelligence the Next Co-Pilot for Primary Care in Diagnosing and Recommending Treatments for Depression?

**DOI:** 10.3390/medsci13010008

**Published:** 2025-01-11

**Authors:** Inbar Levkovich

**Affiliations:** Faculty of Education, Tel-Hai Academic College, Upper Galilee 2208, Israel; levkovinb@telhai.ac.il

**Keywords:** artificial intelligence (AI), primary care, depression, mental health, personalized therapy, psychiatry, ethical considerations

## Abstract

Depression poses significant challenges to global healthcare systems and impacts the quality of life of individuals and their family members. Recent advancements in artificial intelligence (AI) have had a transformative impact on the diagnosis and treatment of depression. These innovations have the potential to significantly enhance clinical decision-making processes and improve patient outcomes in healthcare settings. AI-powered tools can analyze extensive patient data—including medical records, genetic information, and behavioral patterns—to identify early warning signs of depression, thereby enhancing diagnostic accuracy. By recognizing subtle indicators that traditional assessments may overlook, these tools enable healthcare providers to make timely and precise diagnostic decisions that are crucial in preventing the onset or escalation of depressive episodes. In terms of treatment, AI algorithms can assist in personalizing therapeutic interventions by predicting the effectiveness of various approaches for individual patients based on their unique characteristics and medical history. This includes recommending tailored treatment plans that consider the patient’s specific symptoms. Such personalized strategies aim to optimize therapeutic outcomes and improve the overall efficiency of healthcare. This theoretical review uniquely synthesizes current evidence on AI applications in primary care depression management, offering a comprehensive analysis of both diagnostic and treatment personalization capabilities. Alongside these advancements, we also address the conflicting findings in the field and the presence of biases that necessitate important limitations.

## 1. Introduction

Depression is a leading cause of disability worldwide, affecting over 300 million individuals and imposing a significant burden on the healthcare system [1]. The diagnosis of depression primarily relies on two key classification systems: the Fifth Edition of the *Diagnostic and Statistical Manual of Mental Disorders* (DSM-5) [2] and the International Classification of Diseases, 11th Revision (ICD-11) [3]. In the DSM-5, Major Depressive Disorder (MDD) is defined as the presence of at least five symptoms over a two-week period, with at least one being either depressed mood or loss of interest/pleasure. Additional symptoms included significant changes in weight, sleep disturbances, psychomotor alterations, fatigue, feelings of worthlessness, impaired concentration, and recurrent thoughts of death [2]. The DSM-5 delineates depressive disorders into subtypes such as Major Depressive Disorder (single or recurrent episodes), Persistent Depressive Disorder (Dysthymia), Premenstrual Dysphoric Disorder, Substance/Medication-Induced Depressive Disorder, and Depressive Disorder Due to Another Medical Condition [2]. ICD-11 similarly classifies depressive disorders but introduces unique distinctions. It identifies single-episode depressive disorder, recurrent depressive disorder, and persistent depressive disorder (dysthymia) while emphasizing severity levels (mild, moderate, or severe) and the potential presence of psychotic symptoms [3]. Both frameworks recognize specifiers for depression, including seasonal patterns, peripartum onset, and anxiety distress, highlighting the heterogeneity of depressive presentations [2,3].

These diagnostic frameworks guide clinical practice in primary care settings where depression presents significant challenges. The condition often goes unrecognized or is inadequately treated, with studies indicating that only approximately 50% of cases are correctly identified [4,5]. This underdiagnosis is attributed to various factors, including time constraints during consultations, overlapping symptoms with other conditions, and limited mental health training among primary care providers [6,7]. These challenges highlight the need for innovative solutions to improve depression detection and management in primary care settings.

AI offers promising solutions to these challenges by enhancing diagnostic accuracy and personalizing treatment plans [8,9]. AI algorithms can analyze extensive patient data, including medical history and behavioral patterns, to detect early signs of depression that may be overlooked in traditional assessments [10]. AI can also assist in tailoring therapeutic interventions by predicting the effectiveness of various treatments based on individual patient characteristics, thereby optimizing outcomes and improving the efficiency of primary care services [11,12].

The aim of this theoretical review is to examine the role of artificial intelligence in depression care within primary healthcare settings, focusing on two main objectives: (1) analyzing AI’s potential in enhancing depression diagnosis and screening processes and (2) evaluating AI’s contribution to treatment personalization. By synthesizing the current evidence, this review explores both the opportunities and limitations of integrating AI tools in primary care mental health services.

## 2. The Role of AI in Diagnosing Depression

For individuals with depression, primary care providers frequently serve as the initial point of contact with the healthcare system and consequently play a crucial role in initiating appropriate therapeutic interventions [13]. AI systems analyze extensive datasets, including electronic health records and behavioral patterns, to identify early warning signs of depression [14]. For example, machine learning algorithms can process vocal sentiments, facial expressions, and digital activities to detect subtle symptoms. These capabilities are particularly valuable in primary care, where such tools can complement time-limited consultations [11]. Early diagnosis is critical in primary care to prevent the worsening of depressive episodes [15]. AI-powered tools provide real-time risk stratification, helping clinicians prioritize high-risk patients [10].

Recent advancements in AI have introduced sophisticated alternatives to traditional diagnostic methods through the analysis of user-generated text data [16]. Empirical research has established robust correlations between linguistic patterns and mental health status, particularly in the detection of depression [17]. AI algorithms can analyze various forms of written expressions, from social media posts to personal narratives, thereby revealing potential indicators of depressive symptoms. For instance, Havigerová et al. [17] demonstrated that natural language processing techniques can identify depression risk factors in informal writing, such as holiday descriptions. Building on this foundation, De Choudhury et al. [18] developed an innovative detection system employing dictionary-based text analysis to identify depression-associated linguistic markers, thus providing a more objective alternative to conventional self-report measures. Contemporary research by Squires [19] further expanded these capabilities, highlighting the efficacy of advanced algorithms in detecting depressive symptoms through social network content analysis, thereby offering clinicians additional tools for early identification and intervention. AI-based textual analysis relies on advanced computational methods, such as Support Vector Machines (SVMs) and Artificial Neural Networks (ANNs), for detecting depression [20]. SVMs transform text into high-dimensional feature spaces to identify subtle linguistic indicators of depressive symptoms, whereas ANNs process complex patterns to reveal deeper relationships between language use and mental health [20]. These techniques enhance the analysis of patient narratives and clinical notes and offer diagnostic insights that complement traditional assessments [17,18,19,20,21].

AI models are trained on multimodal data such as clinical notes and brainwave patterns to enable the development of personalized assessments that align with the evaluations of mental health professionals [14,15]. A comparative study analyzing the abilities of large language models (LLMs) found that advanced models like ChatGPT-4, Claude, and Bard demonstrated strong alignment with mental health professionals in assessing prognosis and treatment recommendations [22]. These AI systems consistently recommend combined psychotherapy and antidepressant treatment approaches while projecting positive outcomes with professional intervention. Furthermore, AI-driven models have been developed to assess the prognosis of depression, offering insights that align closely with mental health professionals’ evaluations, thereby complementing clinical decision making [22].

Primary care has expressed ambivalent feelings regarding the implementation of depression screening and AI, balancing the acknowledgment of their potential benefits with concerns about their implications. A qualitative study examining primary care highlighted the advantages of screening, such as improved detection of undiagnosed cases, reduced workload, and enhanced communication [23]. However, concerns have been raised about potential harm to the doctor–patient relationship and discomfort caused by screening questions, emphasizing the need for balanced implementation in primary care [23].

## 3. AI in Personalizing Treatment for Depression

Personalization is an effective treatment for depression, and AI algorithms in primary care can predict patient responses to pharmacological and psychotherapeutic interventions based on individual characteristics [24]. This reduces trial-and-error periods and minimizes delays in symptom relief [25]. Recent research comparing ChatGPT-4 and family doctors has found that AI recommendations for treating major depression were consistent with clinical guidelines, demonstrating superior precision in treatment tailoring and showing no gender or socioeconomic bias [8]. Moreover, AI can account for patient preferences, such as comfort with certain therapeutic modalities or concerns about potential side effects, ensuring that treatment plans are both evidence-based and patient-centered. For instance, machine learning algorithms can predict which patients are more likely to respond to cognitive behavioral therapy (CBT) compared to pharmacological interventions, thereby improving the precision of treatment allocation [25].

Studies have shown that LLMs significantly improve diagnostic precision and enhance therapeutic chatbot functionality [26,27]. A randomized controlled trial evaluating an AI platform showed that patients receiving AI-supported therapy had higher session attendance (67% vs. 41%) and greater symptom reduction in depression (34% vs. 20%) and anxiety (29% vs. 8%) [27]. AI-powered personalization also plays a pivotal role in optimizing therapeutic outcomes. Reinforcement learning models have been employed to dynamically adjust treatment plans based on real-time patient responses, ensuring that interventions remain adaptive to evolving clinical needs [28].

**Figure 1:**
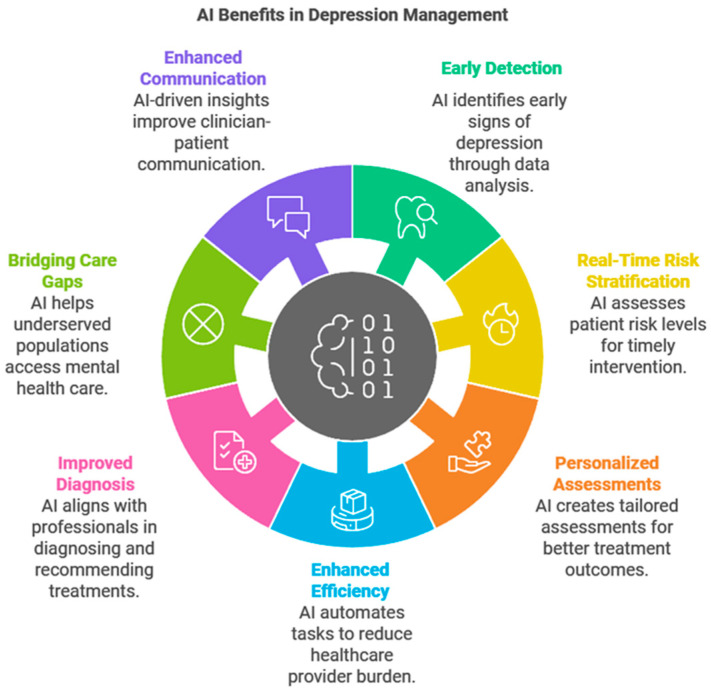
Key AI benefits and applications in depression management.

The benefits of personalized care extend beyond clinical outcomes. For patients, tailored strategies foster empowerment and trust in the treatment process. For providers, AI tools reduce the cognitive burden by synthesizing complex datasets into actionable insights, allowing a focus on therapeutic alliances and nuanced patient concerns [29]. Furthermore, personalization mitigates overmedication risks and unnecessary treatment, thereby promoting cost-effective delivery of care (Figure 1).

## 4. Limitations of AI Integration

Despite the promising potential of AI in mental healthcare, significant challenges remain in ensuring its reliability and generalizability across diverse populations [21,30]. One major issue is the variability in AI model performance when applied to different demographic groups [31,32]. Many AI tools are trained on datasets that do not adequately represent the diversity of global populations, leading to models that perform well in some contexts but fail in others [26]. For example, a model trained primarily on data from high-income countries may underperform when applied to low-resource settings, where healthcare infrastructure and patient demographics differ substantially [33].

This lack of generalizability is further exacerbated by differences in the cultural perceptions of mental health and diagnostic criteria, which can vary widely between regions [34]. AI tools that rely on standardized symptom checklists may fail to account for culturally specific manifestations of depression, such as somatic symptoms, which are more prevalent in some populations [34]. AI tools that rely on standardized symptom checklists may fail to account for culturally specific manifestations of depression. For example, one study observed that depressed individuals from collectivist cultures, such as Chinese communities, are more likely to exhibit somatic symptoms, downplay emotional distress, and suppress emotional expression [35]. Similarly, in Latinx populations, somatic symptoms are often influenced by cultural and linguistic factors, serving as a socially acceptable way to express psychological distress and daily stressors [36]. A cross-cultural study on depression found that East Asian participants reported more somatic symptoms, such as appetite changes, while Latin Americans showed low self-esteem, likely influenced by cultural values tied to collectivism [37]. Consequently, ensuring the global applicability of AI systems requires rigorous testing and adaptation across diverse healthcare settings. Moreover, collaboration with local stakeholders, including clinicians and policymakers, is essential for tailoring these tools to the unique needs of each region [38].

Another critical challenge in the integration of AI into mental healthcare is the presence of biases in datasets and algorithms [39]. Bias can arise from multiple sources, including historical inequalities reflected in the training data, underrepresentation of minority groups, and systemic healthcare disparities [40].

Ethical considerations are paramount when deploying AI for mental health. Issues such as data privacy, informed consent, and transparency in decision making must be carefully addressed to maintain patient trust [41]. Furthermore, the potential for over-reliance on AI tools raises concerns regarding the dehumanization of care [42]. Although AI can augment clinical decision making, it cannot replace the empathy and nuanced understanding that clinicians bring to therapeutic relationships. Overreliance on AI could lead to a reduction in critical thinking among healthcare providers and diminish the quality of patient-centered care [43] (Table 1).

## 5. Implications for Primary Care in Depression Treatment

The integration of AI into primary care has the potential to transform depression screening, diagnosis, and treatment monitoring, while enhancing the patient–provider relationship in managing depressive disorders [32]. However, its adoption requires careful planning to ensure optimal benefits for both patients with depression and their providers [43,44].

AI tools facilitate data-driven interactions, enabling real-time insights into patient depression risk factors and supporting proactive personalized mental healthcare [11,45]. Predictive analytics enables providers to anticipate deterioration in depressive symptoms, facilitating a shift from reactive to preventive approaches in depression management. This enhances outcomes and fosters trust by delivering tailored interventions [46,47]. However, excessive reliance on AI risks depersonalizes depression care as providers may prioritize algorithmic outputs over meaningful engagement [48]. Balancing AI-driven insights with empathetic patient-centered communication is essential [41,49].

**Figure 2:**
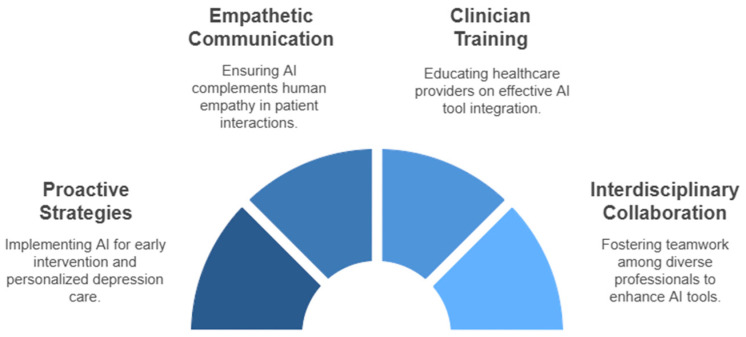
Key implications for AI integration in depression care within primary care.

Effective integration depends on equipping clinicians with AI literacy to interpret outputs, understand limitations, and incorporate clinical judgment in depression care [50,51,52]. Training programs should familiarize providers with depression risk prediction models and mental health decision support systems while promoting critical evaluation and adaptability in diverse clinical contexts [53]. Collaboration among mental health clinicians, data scientists, and engineers is crucial to optimizing AI tools for practical applications in primary care depression treatment [44] (Figure 2).

## 6. Future Directions in Depression Treatment

Despite the significant advancements in AI for diagnosing and managing depression, its long-term efficacy in primary care remains underexplored [8,9,22]. Future research must focus on validating AI tools in diverse primary care environments characterized by varying patient demographics, resource constraints, and time limitations [30,33].

Key areas for future exploration include the development of culturally sensitive AI models. Many existing algorithms fail to account for cultural differences in mental health manifestations, such as somatic symptoms, which are more prevalent in certain populations [22,32]. Moreover, addressing biases in training datasets is essential to ensure equitable care across diverse communities [39,40]. Inclusive datasets and rigorous validation studies are critical for enhancing the reliability of AI applications in underserved and rural areas.

Ethical and regulatory considerations also demand attention. These include ensuring data privacy, obtaining informed consent, and maintaining transparency in AI-driven decisions [31]. Additionally, research should explore the impact of AI on patient trust and the potential for over-reliance among clinicians, which could diminish critical thinking and relational care [43].

**Figure 3:**
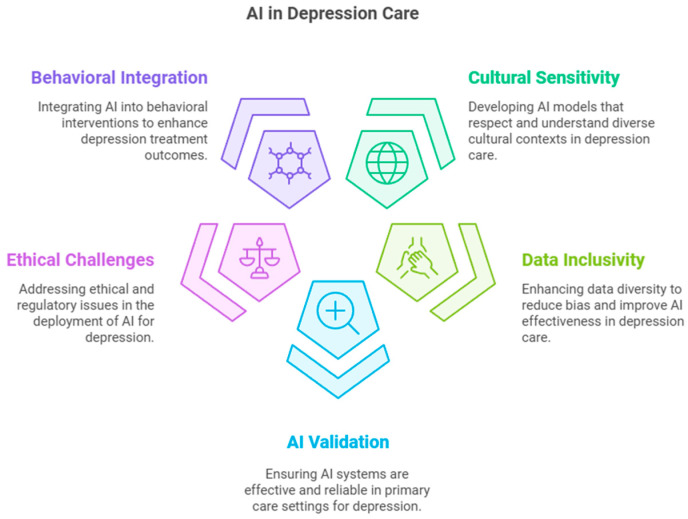
Future directions in depression care within primary care.

AI systems with real-time adaptability, such as reinforcement learning models, hold promise for dynamically adjusting treatment plans based on patient responses, ensuring interventions remain clinically relevant [28]. Expanding AI’s role in integrating behavioral health into primary care, including identifying patients for cognitive–behavioral therapy or lifestyle interventions, represents another frontier for exploration [10,22,48] (Figure 3).

## 7. Discussion

This theoretical review offers several novel contributions to the understanding of AI applications in depression care within primary care settings [32]. The findings highlight both the transformative potential and the significant challenges in implementing AI for depression diagnosis and treatment. In terms of diagnosis, our review revealed that AI tools demonstrate promising capabilities in the early detection and screening of depression [11,12,16,17]. The integration of multiple data sources, from electronic health records to the natural language processing of patient narratives, enables a more comprehensive assessment than traditional methods alone. AI’s ability to detect subtle indicators of depression that might be overlooked in time-constrained primary care consultations is particularly noteworthy [14,18]. However, the accuracy and reliability of these tools vary across different populations and healthcare settings, highlighting the need for broader validation studies [30,31].

Regarding treatment personalization, the evidence suggests that AI can significantly enhance therapeutic decision making [24,25]. The ability to predict treatment responses based on individual patient characteristics represents a major advancement toward precision psychiatry in primary care. The demonstrated success of AI-supported therapy, with improved attendance rates and symptom reduction [27], indicates the potential of AI to augment traditional treatment approaches. Nevertheless, questions remain regarding the long-term effectiveness and optimal integration of these tools in clinical practice [32,44,46,47].

Cultural considerations have emerged as critical factors in the implementation of AI. Our analysis revealed that current AI models often lack cultural sensitivity and may not adequately account for the diverse manifestations of depression across different populations [34]. This limitation is particularly relevant in primary care settings that serve diverse communities, where cultural competence is essential for effective care delivery [35,36,37]. Thus, the ethical implications of AI integration deserve special attention [41]. Although AI offers powerful capabilities for improving care efficiency and accessibility, concerns about data privacy, patient autonomy, and the preservation of human elements in healthcare cannot be overlooked [41,42]. The balance between technological advancement and the maintenance of therapeutic relationships remains a crucial consideration [43,48,49].

Regarding practical implementation, our findings suggest the need for comprehensive approaches addressing several key areas: standardized protocols for AI integration in primary care, healthcare provider training in AI literacy [50,51], the development of culturally adaptive AI models [39], and the establishment of clear ethical guidelines for AI use in mental healthcare [31,45]. Future research should prioritize long-term effectiveness studies in diverse settings, the validation of culturally sensitive models, investigation of optimal AI-clinical expertise integration, cost–effectiveness assessments, and the exploration of patient perspectives regarding AI-assisted care [52,53,54]. These findings collectively suggest that while AI holds significant promise for transforming depression care in primary care settings, its successful implementation requires careful attention to technical, cultural, and ethical considerations [32,44].

## 8. Conclusions

Artificial intelligence represents a groundbreaking advancement in the screening, diagnosis, and treatment of depression, particularly in primary care. By offering precision in detecting depressive symptoms, improving diagnostic accuracy, and enabling personalized treatment plans for patients with depression, AI has the potential to address many longstanding challenges in mental healthcare. However, the integration of AI into healthcare must be approached thoughtfully with attention to ethical considerations, cultural sensitivity, and the preservation of patient-centered care. Although AI can significantly enhance depression assessment and treatment personalization, its successful implementation depends on balancing technological innovation with the relational and humanistic elements of healthcare.

As AI continues to evolve, its role in mental healthcare will undoubtedly expand, offering new opportunities to improve outcomes and bridge gaps in access and equity. Ensuring that these advancements are accessible, equitable, and aligned with the needs of diverse patient populations is critical to their success.

## Figures and Tables

**Table 1:** Limitations in integrating AI into primary healthcare systems.

Key Idea	Description	Proposed Solutions
Limitations in AI Reliability and Generalizability	AI models often perform inconsistently across different demographic groups in primary care settings due to training on non-representative datasets.	Test and adapt AI models across diverse healthcare settings to improve reliability and generalizability in primary care.
Cultural Variability in Depression Care	Cultural differences in mental health perceptions, including depression symptoms such as somatic manifestations, limit the applicability of standardized AI tools.	Collaborate with local stakeholders to tailor AI tools to culturally specific needs and manifestations of depression.
Bias in AI Datasets and Algorithms for Primary Care	Bias arises from historical inequalities, underrepresentation of minority groups, and systemic disparities, impacting fairness and effectiveness in depression care.	Ensure diverse representation in training datasets and address systemic biases to promote fairness in AI algorithms for depression care.
Ethical Considerations in AI for Depression Treatment	Data privacy, informed consent, and transparency in decision making are critical to maintaining patient trust when using AI in depression treatment.	Establish ethical guidelines to protect patient data and uphold transparency and accountability in AI-driven depression treatment.
Risk of Overreliance on AI in Primary Care	Excessive reliance on AI in primary care risks dehumanizing depression treatment, reducing critical thinking among providers, and compromising patient-centered care.	Balance AI insights with clinician empathy and judgment to preserve the human aspect of depression care in primary care settings.

## Data Availability

The data from this manuscript were obtained from publicly available published study results.

## Abbreviations

AI	Artificial intelligence
LLMs	Large language models
CBT	Cognitive behavioral therapy
